# Remedial Treatment of Corroded Iron Objects by Environmental *Aeromonas* Isolates

**DOI:** 10.1128/AEM.02042-18

**Published:** 2019-01-23

**Authors:** Wafa M. Kooli, Thomas Junier, Migun Shakya, Mathilde Monachon, Karen W. Davenport, Kaushik Vaideeswaran, Alexandre Vernudachi, Ivan Marozau, Teddy Monrouzeau, Cheryl D. Gleasner, Kim McMurry, Reto Lienhard, Lucien Rufener, Jean-Luc Perret, Olha Sereda, Patrick S. Chain, Edith Joseph, Pilar Junier

**Affiliations:** a Laboratory of Microbiology, Institute of Biology, University of Neuchâtel, Neuchâtel, Switzerland; b Laboratory of Technologies for Heritage Materials, Institute of Chemistry, University of Neuchâtel, Neuchâtel, Switzerland; c Bioscience Division, Los Alamos National Laboratory, Los Alamos, New Mexico, USA; d Centre Suisse d'Electronique et de Microtechnique, Neuchâtel, Switzerland; e NVENesis, Neuchâtel, Switzerland; f ADMED Microbiologie, La Chaux-de-Fonds, Switzerland; g Haute Ecole Arc Conservation-Restauration, Haute École Spécialisée de Suisse Occidentale, Neuchâtel, Switzerland; North Carolina State University

**Keywords:** *Aeromonas*, artifacts, corrosion, iron, reduction, vivianite

## Abstract

Using bacteria to transform reactive corrosion products into stable compounds represents an alternative to traditional methods employed in iron conservation. Two environmental *Aeromonas* strains (CA23 and CU5) were used to transform ferric iron corrosion products (goethite and lepidocrocite) into stable ferrous iron-bearing minerals (vivianite and siderite). A genomic and transcriptomic approach was used to analyze the metabolic traits of these strains and to evaluate their pathogenic potential. Although genes involved in solid-phase iron reduction were identified, key genes present in other environmental iron-reducing species are missing from the genome of CU5. Several pathogenicity factors were identified in the genomes of both strains, but none of these was expressed under iron reduction conditions. Additional *in vivo* tests showed hemolytic and cytotoxic activities for strain CA23 but not for strain CU5. Both strains were easily inactivated using ethanol and heat. Nonetheless, given a lesser potential for a pathogenic lifestyle, CU5 is the most promising candidate for the development of a bio-based iron conservation method stabilizing iron corrosion. Based on all the results, a prototype treatment was established using archaeological items. On those, the conversion of reactive corrosion products and the formation of a homogenous layer of biogenic iron minerals were achieved. This study shows how naturally occurring microorganisms and their metabolic capabilities can be used to develop bio-inspired solutions to the problem of metal corrosion.

**IMPORTANCE** Microbiology can greatly help in the quest for a sustainable solution to the problem of iron corrosion, which causes important economic losses in a wide range of fields, including the protection of cultural heritage and building materials. Using bacteria to transform reactive and unstable corrosion products into more-stable compounds represents a promising approach. The overall aim of this study was to develop a method for the conservation and restoration of corroded iron items, starting from the isolation of iron-reducing bacteria from natural environments. This resulted in the identification of a suitable candidate (*Aeromonas* sp. strain CU5) that mediates the formation of desirable minerals at the surfaces of the objects. This led to the proof of concept of an application method on real objects.

## Abstract (precis)

Microbiology can greatly help in the quest for a sustainable solution to the problem of iron corrosion, which causes important economic losses in a wide range of fields, including the protection of cultural heritage and building materials. Using bacteria to transform reactive and unstable corrosion products into more-stable compounds represents a promising approach. The overall aim of this study was to develop a method for the conservation and restoration of corroded iron items, starting from the isolation of iron-reducing bacteria from natural environments. This resulted in the identification of a suitable candidate (*Aeromonas* sp. strain CU5) that mediates the formation of desirable minerals at the surfaces of the objects. This led to the proof of concept of an application method on real objects.

## INTRODUCTION

Iron corrosion represents a major problem in man-made ecosystems and causes large economic losses in fields as diverse as water supply, food industries, transport, and cultural heritage (1). While the first three fields are obviously of worldwide importance, the same is the case for the latter, as cultural heritage is a driver of tourism and sustainable development (2). The damage generated by uncontrolled corrosion of archeological objects is a clear example of the negative impact of iron corrosion. Reactive iron oxides and oxyhydroxides are the principal unstable iron corrosion products reported for these objects. Due to their molar volume (higher than that of native iron or iron alloys), these corrosion products generate cracks and irreversible damage of the objects if no intervention occurs (3).

In nature, the phenomenon of iron corrosion is an essential part of the biogeochemical cycling of iron. While aerobic corrosion is considered to be primarily a chemical process, anaerobic corrosion is often related to oxidative and/or reductive bacterial activity (1). Iron is a redox-sensitive element. It oxidizes easily in the presence of oxygen to form insoluble Fe(III) oxides. Iron oxides accumulate in sediments and soils (4), where iron can be remobilized through biotic and abiotic reactions (5, 6). Some bacteria are able to catalyze transformations of iron minerals under oxic and anoxic conditions (4). These bacterial metabolisms can be exploited to transform reactive corrosion products (e.g., lepidocrocite) into stable compounds with low molar volumes (7). This represents a promising biotechnological approach to remediate the problem of iron corrosion.

In two recent studies (8, 9), the feasibility of a biological treatment using bacteria to remediate iron corrosion has been demonstrated. The strategy consists of the reduction of reactive Fe(III) oxides and oxyhydroxides and the formation of biogenic minerals under anaerobic conditions. Initially, a strict anaerobe (Desulfitobacterium hafniense) was used to reduce reactive corrosion products during anaerobic growth. Although reductive dissolution was observed, this approach resulted in the formation of undesirable products. Indeed, in addition to bacterial iron reduction, abiotic reduction caused by the reductant in the medium also occurred and resulted in the formation of sulfur-containing Fe(II) minerals. In addition, the handling of a strict anaerobe and the medium used for reduction were not particularly suitable for application on real artifacts (8). In a follow-up study (9), the bacterium Shewanella loihica was used to stabilize corroded iron coupons, but it required the addition of 1% NaCl, as this bacterium could not reduce iron in the absence of salt. However, the addition of NaCl to the reductive medium is problematic because the presence of chlorides or the formation of chlorine-containing iron oxyhydroxides (e.g., akaganeite) enhances undesirable corrosion processes (10). Hence, there is a need for identifying more-suitable bacterial candidates by selecting facultative anaerobes that could reduce iron in the absence of added chlorinated salts.

Freshwater sediments are a potential source of metal reducers because of the presence of ferric ions as electron acceptors in the form of poorly crystalline Fe(III) oxides (6, 11). In order to select new strains, a screening of bacterial isolates from two Swiss lakes was performed. As a result, two isolates belonging to the genus *Aeromonas* (*Aeromonas* sp. strain CA23 and *Aeromonas* sp. strain CU5) were identified. These facultative anaerobic bacteria were able to reduce soluble and solid Fe(III) phases (12). *Aeromonas* spp. are ubiquitous bacteria associated with aquatic environments (13). Some species are reported to be facultative dissimilatory iron reducers (13). Among the species known to reduce iron, Aeromonas hydrophila is the best-known example (14). However, one risk of an approach starting from the selection of environmental iron-reducing strains is the unintentional enrichment of pathogens. Indeed, *Aeromonas* can be found in many environmental niches (15). Some *Aeromonas* species, such as Aeromonas sobria or Aeromonas salmonicida, are known to be pathogens of aquatic animals and especially of fish. Likewise, A. hydrophila and Aeromonas veronii have been related to human infection (16–18). The goal of this study consisted of improving the development of a biotechnological treatment for the stabilization of corroded iron heritage objects by testing the ability of the two *Aeromonas* isolates to convert reactive iron corrosion products into biogenic Fe(II) minerals. In addition, we investigated the iron reduction mechanism and the potential virulence of the isolates. Finally, we performed tests of an application procedure on real artifacts to demonstrate the validity of the bio-inspired solution proposed here for conservation and restoration of iron artifacts.

## RESULTS AND DISCUSSION

### Biogenic formation of Fe(II) minerals on corroded iron plates.

**FIG 1:**
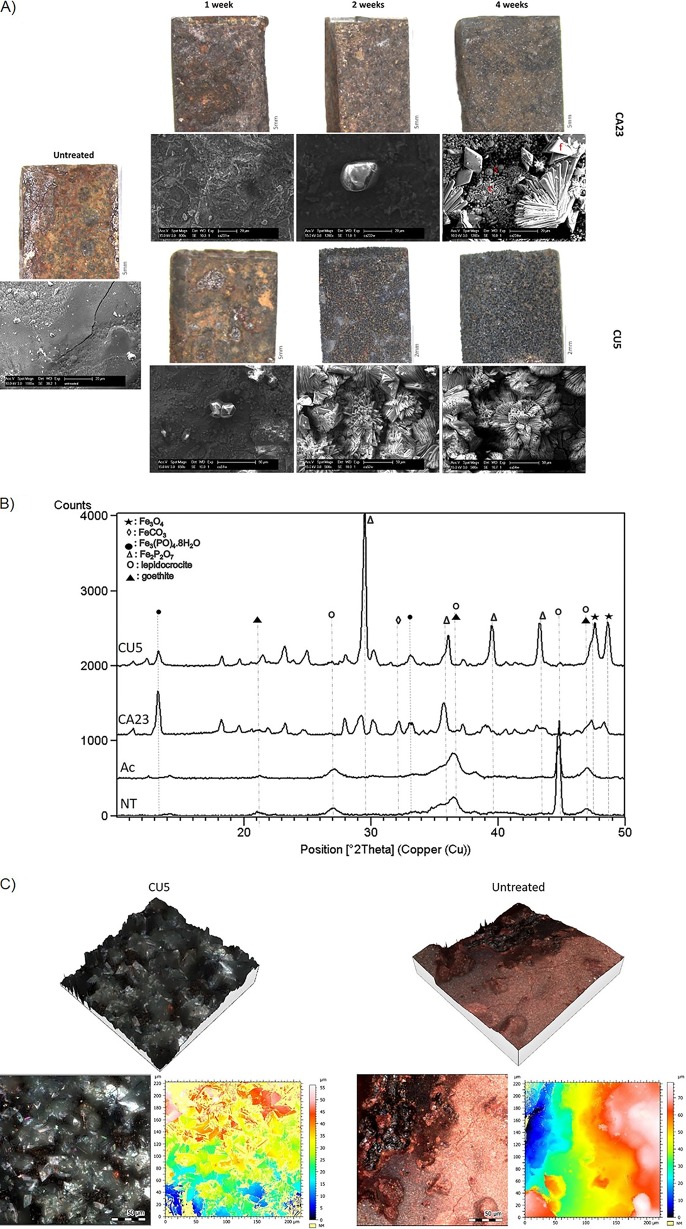
Iron reduction and biogenic mineral formation on corroded iron plates treated with strains CA23 and CU5. (A) Visual appearance (top) and scanning electron microscopy (SEM) images (bottom) of the iron coupons treated with strains CA23 and CU5 after 1, 2, and 4 weeks of incubation compared to an untreated coupon. The red letters f, s, and c indicate foil-, sphere-, and cube-like aggregates, respectively. (B) X-ray diffraction (XRD) diffractograms of corroded iron coupons treated with CU5 and CA23 for 4 weeks, the abiotic controls (Ac), and the untreated coupons (NT). (C) Confocal microscopy images of iron coupons. Left, plate treated with strain CU5 for 4 weeks. Right, untreated plate.

The overall goal of the bio-based treatment is transformation of the reactive corrosion layer on an iron object into biogenic Fe(II) minerals as by-products of the reduction of Fe(III) oxyhydroxides. This should be performed in a solution without NaCl because of the negative effect of chloride ions on the stability of iron objects (10). The results obtained showed that the macro- and microscopic appearances of corroded iron plates treated with the two selected *Aeromonas* strains changed, starting from 2 weeks of incubation with strain CU5 and after 4 weeks of incubation with strain CA23 (Fig. 1A). In the abiotic control treatment (medium without bacteria), no visible change of the iron coupons was observed, suggesting that the solution used for the reduction of reactive Fe(III) oxyhydroxides does not contribute to the transformation of the reactive corrosion layer (see Fig. S1 in the supplemental material). In the plates treated with bacteria, the elemental composition of the surface changed during incubation (Table 1); energy-dispersive X-ray spectroscopy (EDS) analysis showed the presence of phosphorus in the foil-like habit “f” starting from 2 weeks with CU5 and after 4 weeks with CA23 (Fig. 1A). On the corroded iron plates treated with CA23, minerals with cube-like “c” and sphere-like “s” habits were also observed by scanning electron microscopy (SEM) (Fig. 1A). EDS analysis showed the presence of iron, oxygen, and carbon for the “c” habit and of additional sodium for the “s” habit (Table 1).

**TABLE 1:** Atomic percentages of the elements obtained from energy-dispersive X-ray spectroscopy performed on iron coupons after 1, 2 and 4 weeks of treatment with strains CA23 and CU5 and on untreated coupons

Element	Atomic % at week:								
	1		2		4				Untreated iron coupon
	CA23	CU5	CA23	CU5	CA23^*a*^			CU5	
					f	s	c		
C^*b*^	10.33	26.96	27.98	12.96	5.56	25.78	29.73	13.30	18.75
O	23.92	56.05	55.55	57.50	60.63	47.12	45.15	39.26	43.42
Fe	63.42	—^*c*^	—	16.79	21.09	27.09	23.00	30.23	36.28
P	—	—	—	12.75	12.72	—	—	17.21	—
Ca	—	16.99	16.13	—	—	—	—	—	—
Na	—	—	0.34	—	—	—	2.12	—	—
Si	2.32	—	—	—	—	—	—	—	—
Cl	—	—	—	—	—	—	—	—	1.56

a The letters f, s, and c refer to the foil-, sphere-, and cube-like aggregates, respectively, shown in Fig. 1.

b The values from the EDS analysis for carbon (C) should be considered qualitative.

c —, not detected.

X-ray diffraction (XRD) analysis was performed on the iron coupons treated for 4 weeks in order to identify the mineral crystalline phases present on the surface (Fig. 1B). The diffractograms showed the presence of two Fe(II) phosphate minerals [vivianite, Fe_3_(PO)_4_·8H_2_O and Fe_2_P_2_O_7_], as well as magnetite (Fe_3_O_4_). In addition, a Fe(II) carbonate (siderite, FeCO_3_) was detected only on the coupons treated with CA23. The minerals detected are in accordance with the literature reporting that most of the Fe(II) produced by microbial iron reduction precipitates as magnetite, siderite, or vivianite (7). These three minerals were absent in the abiotic controls and on the untreated coupons. The results are consistent with the EDS analysis, even though aggregates composed solely of Fe and O cannot be differentiated through EDS. Moreover, the peaks corresponding to reactive Fe(III) oxyhydroxides (goethite and lepidocrocite), which were present in the diffraction pattern of abiotic controls and untreated coupons, decreased in intensity in coupons treated with strain CA23 and were completely absent in coupons treated with CU5. This result demonstrates the efficiency of the biological treatment for the transformation of reactive corrosion products into biogenic minerals. The effectiveness of this bacterial treatment was confirmed also by confocal microscopy observations (Fig. 1C). The surfaces of biologically treated iron coupons were completely covered by crystals as seen in a 3-dimensional reconstruction. This was also reflected by a change in appearance from a rusty color for the untreated control coupons to a grayish color for the treated ones. Overall, the results of the reduction experiments with the corroded iron plates demonstrate the potential of using these two environmental strains for the development of a bio-based conservation method for corroded iron artifacts.

### Phylogenomic classification of strains CA23 and CU5.

**FIG 2:**
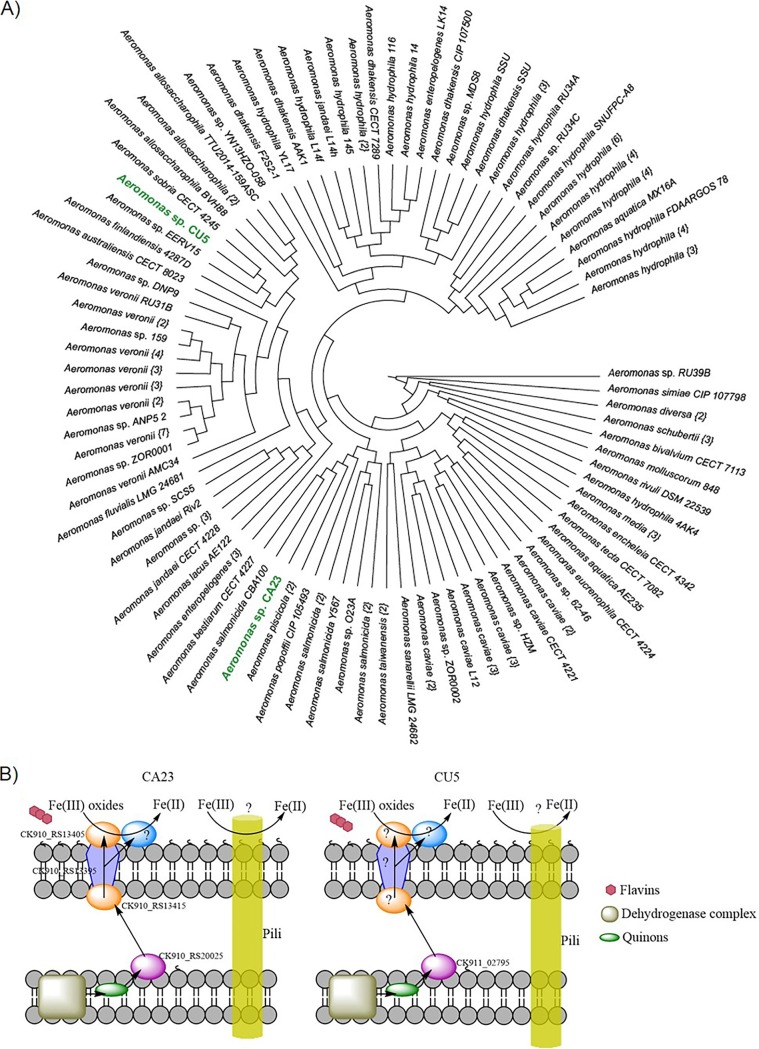
Phylogenomic analyses and potential solid iron reduction pathway of the bacterial strains CA23 and CU5. (A) A cladogram indicating the phylogenetic positions of the two isolates CA23 and CU5 (green) within the genus *Aeromonas*. (B) Homologous genes identified in the genomes of CA23 and CU5, based on the analysis of the genome of A. hydrophila and the iron reduction pathway of S. oneidensis (25).

In order to classify the strains isolated, we used a whole-genome phylogenetic analysis to compare strains CA23 and CU5 with all the available genomes of *Aeromonas* deposited in GenBank (Fig. 2A). The phylogenomic tree shown in Fig. 2 is in general agreement with the current taxonomic classification of species within the genus (19, 20) and further supports the hypothesis that the two isolates belong to two different species. This difference was also confirmed by biochemical tests (see Table S1 in the supplemental material) and average nucleotide identity (ANI) analysis. The ANI value calculated between the two strains was 85.61% (see Fig. S2A in the supplemental material). According to the analysis, strain CA23 could belong to either Aeromonas bestiarum CECT 4227 or A. salmonicida CBA100, given its placement in the phylogenomic tree (Fig. 2A). This was supported by the ANI values, which were greater than 95% in both cases (Fig. S2B). Both A. bestiarum CECT 4227 and A. salmonicida CBA100 are reported to be fish pathogens. The latter was the first Chilean pathogenic isolate to be sequenced, and the analysis of its genome shows a diverse set of mechanisms involved in resistance to multiple antibiotics (21). However, none of these species has been reported to reduce iron. Concerning CU5, the results show that this bacterial strain is closely related to A. sobria CECT 4245 and *Aeromonas* sp. strain EERV15, suggesting that it could belong to the species A. sobria (Fig. 2A and S2C). Although A. sobria is also considered an opportunistic fish pathogen, there is increasing evidence of a large phenotypic diversity within this species (22). Iron reduction for A. sobria has not yet been assessed.

### Electron transport chain for solid iron reduction in CA23 and CU5.

The iron reduction mechanism in *Aeromonas* is less well characterized than the mechanism described for the model iron reducers *Shewanella* and *Geobacter*. In Shewanella oneidensis, the *mtrCAB* operon, as well as the *omcA* and *undA* genes, encode the key proteins involved in iron reduction (23). In A. hydrophila, the mechanism for reduction of Fe(III)-bearing minerals involves a respiratory electron transport chain (ETC) in which reducing equivalents enter via a succinate dehydrogenase and are transferred from quinones and *c*-type cytochromes to a membrane-associated Fe(III) reductase (6). It has been proposed that in A. hydrophila the cluster involved in iron reduction is homologous to the *mtrCAB* operon of S. oneidensis, but no homologs of *omcA*, *undA*, or *mtrDEF* have been identified (24). We analyzed the genomes of CA23 and CU5 to propose a potential ETC for the reduction of iron minerals based on the model of the electron transport to solid iron reported for S. oneidensis (23) and A. hydrophila (24). Through using blastp (identities ranging from 60% to 80%), we proposed a model for CA23 including homologs of the *mtrCAB* complex (gene loci CK910_RS13405, CK910_RS13415, and CK910_RS13395, respectively) and a homolog of *cymA* (CK910_RS20025) (Fig. 2B). Interestingly, in CU5, only a homolog of *cymA* (CK911_02795) and no homologs of *mtrCAB* were identified using blastp (identities below 35%) and Needleman-Wunsch (identities below 20%) searches. This suggests that this bacterium has a different iron reduction pathway. As genes encoding pili have been found to be involved in iron reduction in other bacteria, such as Geobacter sulfurreducens, a search for genes encoding pili was also performed. However, only a homolog of the gene *pilC* in G. sulfurreducens was identified in the genomes of both strains (see Table S2 in the supplemental material).

Because it was previously observed for S. loihica (9) that the production of Fe(II) phosphate minerals in a medium lacking phosphate can be related to polyphosphate accumulation (25), we investigated this trait in *Aeromonas*. Indeed, the genomes of CA23 and CU5, as well as those of A. hydrophila, A. salmonicida, and A. veronii, all possessed two genes coding for polyphosphate kinases and one gene coding for a polyphosphatase (Fig. S2D). These genes are known to be involved in polyphosphate accumulation (26). Interestingly, even though polyphosphate accumulation in *Aeromonas* has been reported (27), especially for A. hydrophila (28), no extensive information on this process could be found, which is surprising because genes involved in polyphosphate accumulation were predicted in several *Aeromonas* genomes.

### Analysis of the pathogenic potential of the strains.

The unintended selection of pathogenic bacteria is a risk when using environmental samples as a source of strains involved in iron cycling. Iron metabolism plays a pivotal role in competition and pathogenicity (29). Therefore, the pathogenic potential of environmental isolates has to be considered during the development of a biological approach to remediate iron corrosion. *Aeromonas* species are autochthonous inhabitants of aquatic environments such as freshwater, brackish water, sewage, and wastewater (16), and some studies have shown that some motile *Aeromonas* species are becoming food- and waterborne pathogens of increasing importance (30). *Aeromonas* species are known as causative agents of a wide spectrum of infections in humans and animals, predominantly amphibians, fish, and reptiles (31). The ability of A. hydrophila, A. caviae, and A. veronii to cause disease can be attributed to a broad range of virulence factors (16). These pathogens also display variable degrees of antimicrobial resistance.

Given the pathogenic lifestyle of the closest relatives to our environmental isolates, we performed several analyses to infer a potential pathogenic lifestyle. First, we considered the resistance of the strains to a series of antibiotics. CA23 and CU5 have the same antibiotic resistance profile (see Table S3 in the supplemental material). They are resistant to some β-lactam antibiotics, such as ampicillin, amoxicillin, and cefalotin, suggesting that they could have a cephalosporinase (a β-lactamase). Indeed, it has been demonstrated that almost all *Aeromonas* species are resistant to ampicillin and cefalotin (30).

Next, we investigated the potential pathogenic lifestyle of the two *Aeromonas* strains by analyzing the repertoire of virulence factors found to be encoded in their genomes. According to the literature, the main pathogenic factors associated with *Aeromonas* spp. include structural features such as pili and flagella, lipopolysaccharides (LPS), and an S-layer. Other extracellular factors, such as iron binding systems (e.g., siderophores), proteases, chitinases, amylases, secretion systems, β-lactamases, hemolysins, and toxins (mainly enterotoxins), also participate in pathogenicity. Indeed, hemolysin and aerolysin are considered the main virulence factors in A. hydrophila, being responsible for hemolytic, cytotoxic, and enterotoxic activities (13, 31). The search for genes coding for potential virulence factors in the genomes of CA23 and CU5 showed that, indeed, these two *Aeromonas* strains possessed most of the genes reported for pathogenicity (Table 2). CU5 appears to have a lesser pathogenic potential, as genes coding for toxins, enterotoxins, and aerolysin were not found in its genome. This tends to favor CU5 for the proposed bio-based treatment to be applied by conservators and restorers.

**TABLE 2:** Gene loci coding for virulence factors found in genomes and RNA-Seq data for CA23 and CU5

Virulence function	CA23^*a*^		CU5	
	Genome	RNA-Seq data	Genome	RNA-Seq data
Adherence and motility				
Pilus assembly, mannose-sensitive hemagglutinin-type pilus, type IV pili	CK910_RS01560-70	NS^*b*^	CK911_RS05025	NS^*b*^
	CK910_RS03095-115	NS^*b*^	CK911_RS08980	NS^*b*^
	CK910_RS07360	NS^*b*^	CK911_RS12050-70	NS^*b*^
	CK910_RS08660	NS^*b*^	CK911_RS14800	NS^*b*^
	CK910_RS08740-45	NS^*b*^	CK911_RS15460-80	NS^*b*^
	CK910_RS11045-65	NS^*b*^	CK911_RS19975-80	NS^*b*^
	CK910_RS13525	NS^*b*^	CK911_RS19750-55	NS^*b*^
	CK910_RS13830	NS^*b*^	CK911_RS19970-80	NS^*b*^
	CK910_RS17960	+^*c*^		
Flagella	CK910_RS19080	+^*c*^		
	CK910_RS20680-740	+^*c*^		
	CK910_RS12690-2765	+^*c*^	CK911_RS03930-60	+^*c*^
	CK910_RS18940	+^*c*^	CK911_RS03990-4030	+^*c*^
	CK910_RS19330-50	+^*c*^	CK911_RS04445-65	+^*c*^
	CK910_RS21170	+^*c*^	CK911_RS10705-85	+^*c*^
			CK911_RS14955-15130	+^*c*^
LPS	CK911_RS08650	NS	CK911_RS20715	NS
	CK911_RS10415	NS	CK911_RS01545	NS
	CK911_RS18840	NS	CK911_RS02440	NS
O antigen	CK910_RS12525	−	CK911_RS10425	NS
	CK910_RS12555	−	CK911_RS10495	NS
			CK911_RS17675	NS
Toxins and extracellular enzymes				
Toxins and enterotoxins	CK910_RS00960	+^*d*^	CK911_RS01110	+^*e*^
	CK910_RS12220	NS		
	CK910_RS13090	NS		
	CK910_RS19835	NS		
	CK910_RS12375	NS		
Hemolysins	CK910_RS09570	NS	CK911_RS00740	NS
	CK910_RS10940	NS	CK911_RS09825	NS
	CK910_RS20415	NS	CK911_RS12215	NS
Aerolysins	CK910_RS02845	NS	−	
Chitinase	CK910_RS00070	NS	CK911_RS00935	NS
	CK910_RS09840	NS	CK911_RS12780	NS
Metalloprotease	CK910_RS10490	NS	CK911_RS00235	NS
	CK910_RS01820	NS	CK911_RS11850	NS
	CK910_RS02990	NS	CK911_RS15345	NS
	CK910_RS07020	NS	CK911_RS18785	NS
			CK911_RS19670	NS
Other proteases	23 loci	NS	20 loci	+^*c*^
Hyaluronidases	CK910_RS20620	−	CK911_RS10170	−
Collagenases	CK910_RS02455	−	CK911_RS14200	−
	CK910_RS20825	−		
Phospholipases	CK910_RS03220	NS	CK911_RS15625	NS
Alpha-amylases	CK910_RS21730	NS	CK911_RS07125	NS
	CK910_RS22255	NS	CK911_RS11695	NS
Iron acquisition				
Siderophore receptors	CK910_RS02730	NS	CK911_RS17235	NS
	CK910_RS15610	NS	CK911_RS18880	NS
	CK910_RS15610	NS	CK911_RS09860	NS
	CK910_RS04745	NS		
Ferric reductases	CK910_RS15615	−	−	
Ferric uptake regulator Fur	CK910_RS20320	NS	CK911_RS09930	NS
Ferrous iron transporters	CK910_RS04080	NS	CK911_RS09200	NS
	CK910_RS13500	NS	CK911_RS16520	NS
Antibiotic resistance				
β-Lactamases	CK910_RS01300	NS	CK911_RS11640	NS
	CK910_RS04830	NS	CK911_RS12920	NS
	CK910_RS11380	NS	CK911_RS17150	NS
Aminoglycoside acetyltransferases	CK910_RS05645	−	CK911_RS20860	−
Phosphotransferase	CK910_RS00245	−	−	
SapABCDF peptide intake transport	CK910_RS02000	NS	CK911_RS05100	NS
	CK910_RS02555	NS	CK911_RS10300	NS
	CK910_RS12175	NS	CK911_RS19460	NS
	CK910_RS15245	NS		
Transport				
Multidrug transporters	14 loci	NS	18 loci	+^*c*^
Major facilitator superfamily transporter	32 loci	−	13 loci	NS
Secretion system	CK910_RS02125-85	+^*c*^	CK911_RS09150	NS
	CK910_RS02175-85	+^*c*^	CK911_RS10720	NS
	CK910_RS03045-80	+^*c*^	CK911_RS11180-11235	NS
	CK910_RS07355	+^*c*^	CK911_RS14805	NS
	CK910_RS08605	+^*c*^	CK911_RS15410-15	NS
	CK910_RS13705	+^*c*^	CK911_RS15445	NS
	CK910_RS14985-15095	+^*c*^	CK911_RS15440	NS
	CK910_RS18505-15	+^*c*^	CK911_RS20035	NS
	CK910_RS21330-40	+^*c*^		

a NS, not significant; +, found; −, not found.

b Only type IV pili.

c Downregulated.

d Antitoxin, upregulated.

e Antitoxin, downregulated.

However, possession of this arsenal of pathogenicity factors does not imply a pathogenic lifestyle. For example, the β-lactamases encoded in our two strains were demonstrated to be functional and to confer resistance to three β-lactam antibiotics *in vivo* (Table S3). However, the same might not be the case for other pathogenicity factors detected in the genome. Therefore, and given the large range of potential virulence factors, we investigated the expression of these genes under the conditions used to convert reactive Fe(III) corrosion products to Fe(II) minerals (see Fig. S3 in the supplemental material). Under these conditions, none of the genes involved in pathogenicity were significantly expressed (https://figshare.com/s/dbf6be9c8286041db24f, https://figshare.com/s/a5cdd9f0de2e04b612b3). Instead, the genes for the type II secretion system (*epsE*), multidrug transporters (*norM*), and antitoxin (*yfjZ*) were downregulated under iron-reducing conditions (the last was upregulated in CA23) (Table 2).

**FIG 3:**
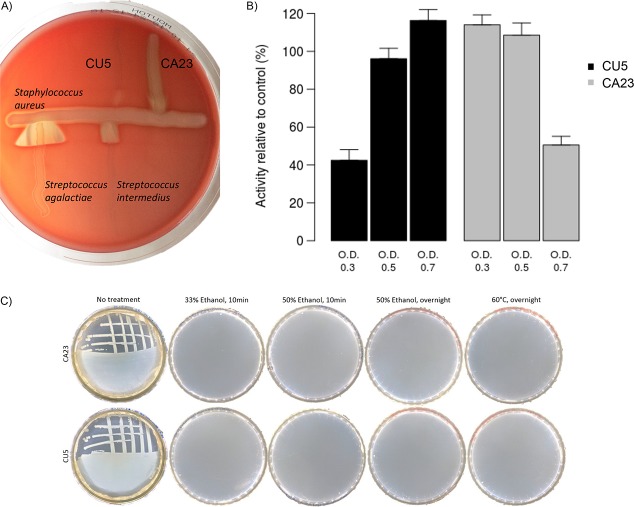
Investigation of the pathogenic potential of *Aeromonas* sp. strains CA23 and CU5. (A) Hemolysis and CAMP factor detection tests using Columbia sheep blood agar. (B) Measurements of the toxicology of *Aeromonas* sp. strains CA23 and CU5 on Caenorhabditis elegans strain N2 (ancestral). Nematode relative activity (compared to the control fed with Escherichia coli OP50 at an OD of 0.7) was measured after 48 h in contact with bacteria at different initial ODs. (C) Images showing the inactivation of the two bacterial strains after treatment with 23% and 35% ethanol (with different exposure times) and heat at 60°C overnight.

Although the results of the transcriptomic analysis would suggest that genes associated with pathogenicity are not relevant in the case of iron reduction, additional analyses are required to establish a potential pathogenic lifestyle for the strains. Therefore, in addition to the investigation of pathogenicity using genomic and transcriptomic approaches, the pathogenic potential of the two isolates was addressed more directly by analyzing the expression of the toxin hemolysin and by evaluating the cytotoxicity of the strains on the nematode Caenorhabditis elegans. *Aeromonas* spp., especially A. hydrophila, A. sobria, and A. salmonicida, are often reported as pathogens and associated with an hemorrhagic effect on warm- and cold-blooded animals (18, 32, 33). Many virulence factors, particularly toxins, have been reported to confer this phenotype (18, 32). Hemolytic activity for strains CA23 and CU5 was tested on sheep blood agar. Strain CA23 was positive for β-hemolysin activity, with a minor presence of a hemolytic synergy with Staphylococcus aureus (Fig. 3A). This synergistic effect is probably due to the presence of CAMP factor (34, 35). In contrast, no hemolysin or CAMP factor effect was observed for CU5 (Fig. 3A). Some studies on A. hydrophila have demonstrated that hemolytic activity in this bacterium requires the simultaneous presence of genes coding for hemolysin and aerolysin (32). Accordingly, *Aeromonas* sp. strain CA23 possesses several genes coding for hemolysins and one for aerolysin, which probably explains the hemolytic activity observed *in vivo* (Table 2). In the case of strain CU5, no genes coding for aerolysin were detected in the genome (Table 2), and this could explain the results obtained (Fig. 3A). In addition, strain CA23 has four genes coding for enterotoxins in its genome, while CU5 possesses only one. This could reinforce the higher pathogenic potential of CA23.

The second *in vivo* test performed measured the cytotoxic effect of both *Aeromonas* strains on C. elegans. The use of nematodes, especially the roundworm C. elegans, is considered an important emerging technique for studying host-pathogen interactions (36, 37). Some studies have reported that the existence of universal virulence factors allows mammal-pathogenic bacteria to cause disease in nonvertebrate hosts (36). Moreover, the presence of conserved signaling pathways could lead to the extrapolation of data from invertebrate studies to humans or other vertebrates (37). Working with the bacterivore C. elegans as a host model has many advantages. To test infection, its normal food source (Escherichia coli OP50) can be replaced (or combined) with the bacterium of interest (36–38). This model has been applied already to investigate the pathogenicity of some *Aeromonas* spp. (36, 38, 39). Adding *Aeromonas* sp. strain CA23 to a bacterial solution to feed C. elegans resulted in a reduced activity of the nematode as a function of the dose (Fig. 3B). At a low dose of CA23 (optical density [OD] of 0.3), C. elegans displayed higher activity (114%) than the control with only regular food bacteria (E. coli OP50). However, nematode activity decreased when CA23 biomass increased (50% at an OD of 0.7). This decrease suggests that CA23 has a deleterious effect on C. elegans. In contrast, the increase in the abundance of *Aeromonas* sp. strain CU5 added to the feeding medium resulted in higher activity of C. elegans (Fig. 3B) (42% at an OD of 0.3 and 116% at an OD of 0.7). This result indicates that the nematode is able to develop and feed on CU5 and suggests that this bacterium does not have a toxicological effect.

All the experiments suggest that CU5 might be a safer option for the development of a bio-based treatment, although entirely ruling out a pathogenic lifestyle by additional experiments is beyond the scope of this study. However, one aspect that must be addressed is the ease of decontamination of the material (for instance, iron artifacts and application matrix) after treatment. Therefore, a test to inactivate the strains by treatment with ethanol and heat was performed. The results showed that even a 10-min treatment with ethanol at a concentration as low as 23% inactivates both strains. The same can be obtained with higher concentrations, longer exposure times, or overnight incubation at 60°C (Fig. 3C), suggesting that a treatment with pure ethanol (to avoid the detrimental effect of water on the surface of an iron artifact) could be considered in actual practice. This indicates that the discarding of these strains can be achieved in a simple way, which is important for biotechnological applications.

### Treatment application on archaeological iron objects.

In order to establish a prototype treatment that would convert the reactive corrosion layer into chemically stable iron minerals, we used a commercial gel to apply bacteria on archaeological iron nails. The preliminary results showed that the treatment was partially successful (see Fig. S4 in the supplemental material); the corrosion layer of the nails was not fully converted, in contrast to the case for the treated iron coupons (Fig. 1). With a real archaeological object, the formation of biogenic iron minerals was observed after 4 weeks with the CA23 bacterium and starting from 2 weeks with the CU5 bacterium. These minerals were composed mainly of O, C, and P according to EDS measurements. Fourier transform infrared spectroscopy (FTIR) analyses were performed to identify the minerals formed. The results showed typical vibration bands of proteins (amide I peak around 1,640 cm^−1^ and amide II peak around 1,420 cm^−1^) indicating the presence of bacteria. The peaks between 1,040 and 940 cm^−1^ correspond to the phosphate absorbance region (40, 41), which confirmed the formation of iron phosphates. Absorbance bands corresponding to the coordinated water associated with iron phosphates could be observed at 3,161 and 795 cm^−1^, which indicates that these minerals have a crystalline phase (40, 41). According to the FTIR spectrum, the biogenic minerals belonged to the vivianite-related group (41).

**FIG 4:**
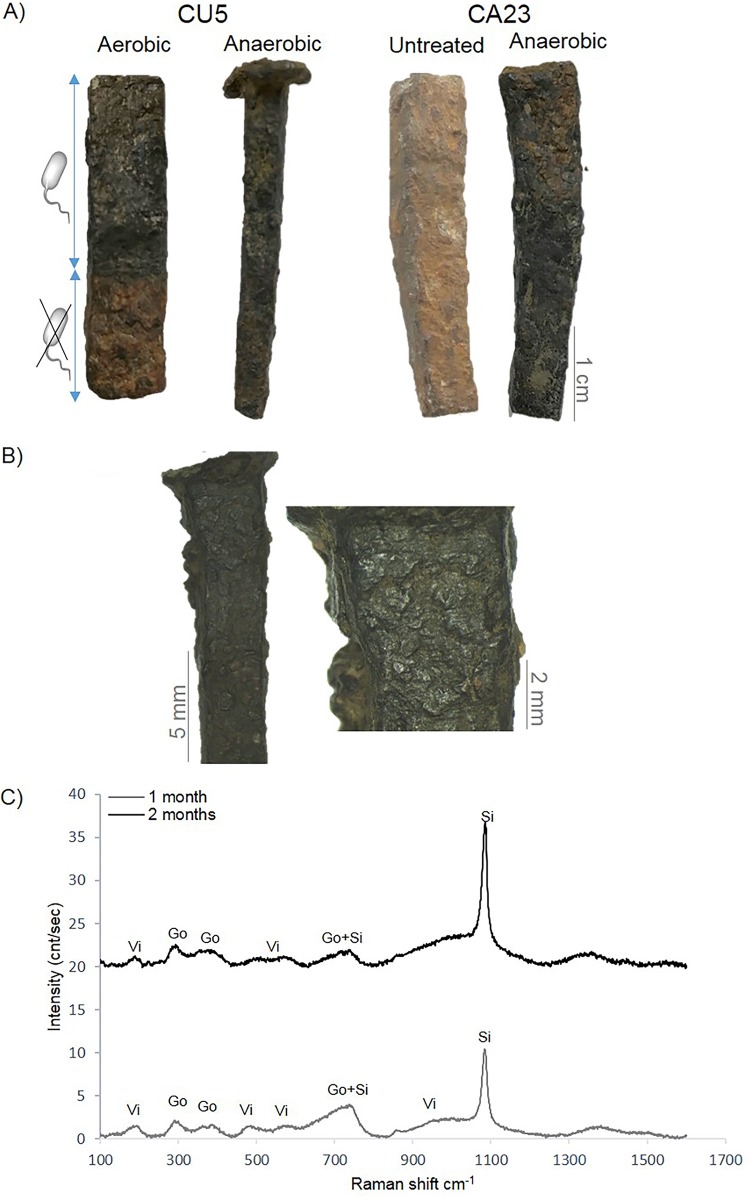
Optimized bacterial treatment of archaeological iron objects. (A) Visual aspect of the iron objects treated for 1 month. Left, iron artifacts partially treated with the bacterium CU5 under aerobic and anaerobic conditions and having their bottom parts used as abiotic controls (gel delivery system without bacteria). Right, a full iron object treated with CA23 under anaerobic conditions in comparison to untreated nails. (B) Microscopic images of an archeological nail treated for 2 months under anaerobic conditions with CU5. (C) Raman spectra obtained from the surfaces of the treated archaeological objects after 1 (gray line) and 2 (black line) months of treatment with CU5 under anaerobic conditions. Corrosion compounds were identified as goethite (Go), vivianite (Vi), and siderite (Si).

To optimize the treatment, we performed a second test by increasing the number of bacterial cells and the incubation time and by treating archaeological iron objects both aerobically and anaerobically. The results are shown in Fig. 4. The increased concentration of bacterial cells had a positive effect on the performance of the treatment, especially compared with the first attempt (Fig. S4). In this case, the treatment using CA23 or CU5 was successful. The corroded archaeological objects were completely covered by mineral compounds, providing a velvety texture and gray-black color to their surfaces (Fig. 4A and B; see Fig. S5A in the supplemental material). This result is not due to the chemical composition of the delivery system, as no change in the visual aspect was observed in the controls without bacteria (Fig. 4A). In order to determine the nature of these gray-black compounds, Raman spectroscopy was performed (Fig. 4C). Vivianite (shifts at 187, 192, 480, 954, and 1,001 cm^−1^) and siderite (shifts at 734, 736, 1,083, and 1,087 cm^−1^) were detected only in the samples treated with CU5 for 1 and 2 months, in comparison to the untreated archaeological iron nails, which have been previously characterized using Raman microscopy (8). The same was the case for the abiotic control (Fig. S5B). These Fe(II) minerals were also present in the treated iron coupons described above (Fig. 1B). Likewise, Raman analysis showed that vivianite was less abundant than siderite in the 2-month samples. Goethite and lepidocrocite (reactive corrosion products) were completely transformed, as lepidocrocite was detected only in the untreated and abiotic control samples, while weak peaks corresponding to goethite were detected mainly in the 1-month samples. Even though partial reoxidation of the treated surfaces was observed after 48 h of air exposure, incubation under aerobic or anaerobic conditions does not seem to influence the efficiency of the treatment, which is positive because an aerobic treatment is easier for application.

There is a growing interest in the synthesis of inorganic components by biological systems in processes that are respectful of the environment (3). As an example, bacterial iron reduction has been used as a low-cost and environmentally friendly biotechnological approach for iron extraction from ores (7). In the cultural heritage field, exploiting specific bacterial abilities for the transformation of unstable iron corrosion products into more stable iron minerals represents a promising bio-based approach to traditional conservation and restoration methods (8). Stepwise improvement of the treatment is leading to a biotechnological application. The first treatment employed a strict anaerobic bacterium growing in a complex medium, which renders optimization challenging (8). In particular, even though the visual characteristics and the composition of the corrosion layers were transformed, the production of undesirable products and abiotic reduction were also observed. Further optimization using the facultative anaerobe S. loihica facilitated the potential up-scaling of the process, but the presence of chloride ions in the iron-reducing medium, which are reported to enhance corrosion on archaeological iron objects (10), is incompatible with actual practice (9). In this study, we proposed a promising iron stabilization method and tested it on outdoor corroded surfaces, as well as on archaeological objects. This treatment was performed under aerobic conditions, without the addition of salt, and the macroscopic aspect of the objects improved due to the transformation of the unstable corrosion layers into mainly siderite and vivianite. The treated objects had a color that agrees with the aesthetic criteria that are conventionally applied in iron heritage conservation. Nevertheless, an improvement of the long-term stability of these Fe(II) minerals that are formed and an assessment of their effective protectiveness against further corrosion should be considered in the future. In addition, more stringent tests are needed to verify a nonpathogenic lifestyle of the strains selected. CU5 appears to be more promising than CA23, based on its gene content and the tests performed in this study. Further investigation of the long-term stability and additional pathogenicity tests with higher organisms are important in order to convert the proposed treatment into a real *in situ* practice for conservators and restorers.

## MATERIALS AND METHODS

### Bacterial strains and growth conditions.

Bacterial isolates from Lake Cadagno and Lake Neuchâtel (Switzerland) were used in this study. Regular cultivation was performed aerobically using Luria-Bertani (LB) medium (1% tryptone, 0.5% yeast extract, and 1% NaCl) at pH 7 and 30°C under agitation at 130 rpm.

### Iron samples.

The corroded iron coupons (10 by 10 by 2 to 3 mm) were cut from iron plates of 50 by 50 by 2 to 3 mm. The plates were exposed for 1 year to an outdoor marine environment (French Institute of Corrosion, Brest, France). The orientation and position of the plates during exposure were selected according to the ISO 9223 standard. A corrosion layer composed of lepidocrocite and goethite was formed and characterizes the iron coupons. The archaeological iron nails were excavated from a chalky soil in Champagne region, France, and dated from the late Roman period (3rd century AD).

### Chemical matrix for solid Fe(III) reduction.

Iron reduction and biogenic mineral formation with the coupons as a solid iron source were performed in a matrix composed of 20 mM piperazine-*N*,*N′*-bis(2-ethanesulfonic acid) (PIPES), 5 mM sodium lactate, and 50 mM NaHCO_3_. For the sterilization of the iron coupons, autoclaving was avoided because further corrosion was observed under both oxic and anoxic conditions. Instead of autoclaving, coupons were washed with 70% (vol/vol) ethanol and dried under UV exposure for 1 h for each side. A control for this sterilization method was performed by incubating UV-sterilized plates on solid LB medium under oxic and anoxic conditions, which showed no bacterial growth after 1 week of incubation.

### Iron reduction of solid Fe(III).

In order to decouple growth from iron reduction, a culture of 50 ml of CA23 and CU5 was prepared by cultivating the bacteria under aerobic conditions in LB medium overnight at room temperature. Cell biomass was then collected by centrifugation (1,700 × *g* for 10 min), and the pellet was washed with 20 mM PIPES solution. The pellet was resuspended in the same solution and added to the matrix containing iron. To evaluate iron reduction, samples were collected every 2 h for 24 h. The production of iron minerals was evaluated after 1, 2, and 4 weeks of incubation at room temperature under agitation (120 rpm). Experiments were performed in triplicates, including abiotic controls (without bacteria).

### Measurements of Fe(II) concentration.

To measure the reduction of Fe(III) into Fe(II), the ferrozine assay was performed following a protocol modified from that of Bell et al.(42). First, 100 μl of 5 M HCl was added to 900 μl of collected samples and stored at 4°C. The samples were centrifuged for 1 min at 6,700 × *g*, and then 100 μl of the supernatant was taken and 900 μl of ferrozine solution (0.1% ferrozine in a 100 mM HEPES solution at pH 7) was added. The Fe(II) concentration was determined by measuring the absorbance at 562 nm with a UV-visible spectrophotometer (Thermo Fisher Scientific Genesis 10s). A calibration curve was obtained using serial dilutions of 1 mM ferrous ammonium sulfate solution in acidic MilliQ water (pH 2).

### Development of a user-friendly treatment of archaeological iron nails.

For this treatment, we tested the effect of the bacteria CA23 and CU5 on the stabilization of archaeological iron nails in a gel delivery system. Before being in contact with the bacteria, the nails were sterilized using 70% (vol/vol) ethanol and placed under UV light for 1 h. Phytagel (Sigma-Aldrich), was chosen as the delivery system. The gelling agent in its initial powder format was sterilized under UV light for 30 min before being mixed with the bacterial culture.

For each nail, 100 ml of overnight cultures of CA23 and CU5 (at room temperature) with ODs (measured at 600 nm) of 1.080 and 1.027, respectively, was centrifuged at 4,000 rpm for 10 min. The pellet was washed twice with 20 mM PIPES solution. The pellet was mixed with 1 g of the gelling agent, and 1 ml of 1 M sodium bicarbonate and 0.5 ml of 1 M sodium lactate were added to this gel mixture. The bacterium-gel system was applied to the nails, which were then covered with cellophane and stored at room temperature. Two conditions were tested: incubation under oxic conditions (the nails were placed in petri dishes) and under anoxic conditions (using Anaerocult A bags [Merck]). To test the treatment’s efficiency and according to the results obtained with the iron coupons, the treatment was stopped after 1 and 2 months for each bacterium. The abiotic control consisted of 2 g of gelling agent mixed with 10 ml of 20 mM PIPES solution amended with 5 mM sodium lactate and 50 mM sodium bicarbonate. The sterility of the controls after treatment was not verified. However, gel without bacteria added did not change in color, while the gel mixed with bacteria turned black after the incubation period.

### Analytical techniques.

**(i) SEM-EDS analyses.** For scanning electron microscopy coupled with energy-dispersive X-ray spectroscopy (SEM-EDS) analyses, the iron coupons were washed twice with deionized water and 70% (vol/vol) ethanol before being mounted on stubs using carbon conductive tape without gold sputtering. To avoid humidification, the stubs were stored in desiccators with silica gel. A Philips ESEM XL30 FEG environmental scanning electron microscope equipped with an energy-dispersive X-ray analyzer was used. The samples and coupons were observed in secondary electron mode at an acceleration potential of 10 to 25 keV and with a 10-mm working distance.

**(ii) XRD analyses.** X-ray diffraction (XRD) analyses were performed on untreated, abiotic control iron coupons and on iron coupons treated for 1 month. The preparation of the iron coupons was the same as for SEM-EDS analyses. High-quality data q-2q (bulk) measurements were performed with a PANalytical X’Pert Pro MPD diffractometer equipped with a fast-linear detector and sample spinner. The measurement time was 60 h to acquire high statistics for better signal-to-noise ratio. The sensitivity of the XRD phase identification is about 3% by weight. The ICSD database was used for the identification of siderite (98-004-6078), vivianite (98-004-6142), and magnetite (98-001-1782).

**(iii) Confocal microscopy.** A three-dimensional (3D) configuration of the iron coupons was reconstructed from a scanning of the surface made at a magnification of ×50 with its corresponding color-coded height map. The images were taken using a Smartproof 5 Zeiss confocal microscope (aperture correlation microscopy) with Zeiss Efficient Navigation (ZEN) and Confomap software.

**(iv) Raman spectroscopy analysis.** Nondestructive Raman spectroscopy was performed directly on the samples to define the molecular compositions of the corrosion layer before and after bacterial treatment. The analysis was carried out with a Horiba-Jobin Yvon Labram Aramis microscope equipped with an Nd:YAG laser of 532 nm at a power lower than 1 mW. The spectral interval analyzed was between 100 and 1,600 cm^−1^. Single-points analyses were carried out with the following conditions: 400-μm hole, 100× LWD objective, D2 filter, 100-μm slit, and 5 accumulations of 100 s. The spectra recorded were corrected (automatic baseline correction) using LabSpec NGS spectral software. Reference spectra were used for identifying the compounds present.

**(v) FTIR analyses.** Fourier transform infrared spectroscopy (FTIR) measurements were performed on the minerals formed on the surfaces of the nails. An iS5 Thermo Fisher Scientific spectrometer with a diamond attenuated total reflectance (ATR) crystal plate (iD5 ATR accessory) was used. All spectra were acquired in the range 4,000 to 650 cm^−1^ at a spectral resolution of 4 cm^−1^. A total of 32 scans were recorded and the resulting interferograms averaged. Data collection and postrun processing were carried out using Omnic software.

### Molecular methods.

**(i) RNA extraction and rRNA removal.** CA23 and CU5 were cultivated on LB medium and then transferred in the same matrix used for solid iron reduction as described above. To identify the genes involved in solid iron reduction, two conditions were tested: with or without iron coupons. The experiment was performed in triplicates. Sampling of 1 ml from each replicate was achieved before inoculating the cells into the matrices at 0, 12, and 24 h of incubation. After sampling, bacterial cells were stored at −80°C. Prior to RNA extraction, samples were thawed on ice and then briefly centrifuged (10,000 × *g* for 1 min). RNA extraction was done using a Zymo Research Direct-zol kit with TRIzol reagent according to the manufacturer’s instructions. A Ribo-Zero magnetic kit for Gram-negative bacteria (Illumina) was used for rRNA removal, according to the manufacturer’s instructions. The concentration of RNA from the extraction was considered to calculate the amounts of reagents used for the preparation of the magnetic beads and for the treatment of total RNA with Ribo-Zero rRNA removal solution (4 μl rRNA removal solution for 0.25 to 1 μg total RNA per reaction). Samples in Ribo-Zero rRNA removal solution were incubated at 68°C for 10 min, followed by 5 min of incubation at 20°C. To remove rRNA, the Ribo-Zero mix incubated previously was mixed with the prepared magnetic beads and placed at room temperature for 5 min and then at 50°C for 5 min. The rRNA-depleted samples were purified using the RNeasy MinElute kit (Qiagen). The final mRNA eluted was quantified using the Qubit-IT RNA HS assay kit (Thermo Fisher Scientific) and Bioanalyzer RNA 6000 Pico kit (Agilent).

**(ii) Illumina indexed library preparation.** The preparation of indexed libraries from RNA and sequencing was performed using Illumina’s ScriptSeq v2 sample preparation kit. As less than 40% of the RNA obtained was more than 600 nucleotides (nt) long, no fragmentation was done. Nevertheless, a maximum of 5 μg of RNA was mixed with 2 μl of cDNA primer (200 μM), incubated at 65°C for 5 min on a thermal cycler, and then placed on ice (1 μl of fragmentation solution was added after incubation according to the manufacturer’s instructions). Three microliters of the cDNA synthesis premix obtained previously was mixed with 0.5 μl dithiothreitol (DTT) (100 mM) and 0.5 μl StarScript reverse transcriptase. The reaction mixture was incubated at 25°C for 5 min and at 42°C for 20 min and then held at 37°C. Next, 1 μl of finishing solution was added, and the reaction mixture was incubated at 37°C for 10 min and 95°C for 3 min and held at 25°C. Eight microliters of tagging master mix (7.5 μl terminal tagging premix and 0.5 μl DNA polymerase) was added, and the tubes were incubated at 25°C for 15 min and 95°C for 3 min and held on ice. The purification of the cDNA was performed using Agencourt AMPure XP beads. PCR was performed using Failsafe PCR enzyme mix (Illumina), made by preparing a PCR master mix (25 μl of PCR premix E, 1 μl of forward PCR primer, and 0.5 μl of Failsafe enzyme [2.5 U/μl]) and adding 1 μl of index primer (Script Seq index PCR primers; Illumina). The reaction mixture was incubated in the thermocycler as follows: 95°C for 1 min and then 15 cycles of 95°C for 30 s, 55°C for 30 s, and 68°C for 3 min. The samples were then incubated at 68°C for 7 min and held at 4°C. The PCR product cleanup was done using AMPure XP beads. The validation and the control of the quality of the library were done using the Qubit-IT double-stranded DNA (dsDNA) HS assay kit and BioAnalyzer DNA high-sensitivity kit (Agilent). Accurate quantification of the amplified library products is important for the sequencing results on the Illumina next-generation sequencing platforms. A quantitative PCR (qPCR) (using the Kapa SYBR Fast universal qPCR kit for Illumina libraries) was performed in order to obtain an accurate quantification of the amplifiable molecules in the DNA libraries.

**(iii) Illumina sequencing and RNA data analysis pipeline.** Libraries were sequenced on an Illumina NextSeq 500 instrument, using the NextSeq sequencing kit HO 300 cycles (Illumina) according to the manufacturer’s instructions (paired-end sequencing mode). The raw reads of transcriptome sequencing (RNA-Seq) data were processed using Pipeline for Reference-Based Transcriptomics (PiReT) software (https://github.com/mshakya/PyPiReT). Briefly, the reads were aligned and assigned to the reference genomes using HISAT2 v2.05 (43) (version information is available in the github repository). Aligned reads were then analyzed and assigned to individual genes according to the genome annotations (GenBank accession no. GCF_002587065.1 and GCF_002586745.1 for CA23 and CU5, respectively) using featureCounts v1.5.2 (44). The normalized read counts for each gene (reads per kilobase per million [RPKM]) were calculated using the R software, package edgeR v3.14.0.

Graphical representations of differential gene expression data obtained from pairwise comparison (volcano plots, principal-component analysis [PCA], and heat maps) were created using R software (45) including the ggplot 2 package (46).

**(iv) Genomic analysis for the genes involved in solid iron reduction, polyphosphate accumulation, and virulence.** Genomic analysis was performed in order to identify the potential genes involved in iron reduction in CA23 and CU5. To do so, we extracted the sequences of the proteins involved in solid iron reduction in the model bacterium S. oneidensis MR-1 (accession no. NC_004347.2) from NCBI (MtrA, NP_717386.1; MtrB, NP_717385.1; MtrC, NP_717387.1; OmcA, NP_717388.1; and CymA, NP_720107.1) as well as the homologs of MtrCAB in A. hydrophila ATCC 7966 (AHA_2765, YP_857273.1; AHA_2766, YP_857274.1; and AHA_2764, YP_857272.1), and we performed a similarity search against the proteomes of CA23 and CU5 (NZ_CP023818.1 and NZ_CP023817.1, respectively) using BLAST package version 2.7.1 (blastp, blastp suite, blastn, and tblastn) (47) with default settings and the Needleman-Wunsch algorithm (48) in its implementation from EMBOSS package version 6.6.0.0 (needle program) (49). The iron reduction pathways of both CA23 and CU5 were drawn using ChemDraw Professional [version 16.0.0.82(68)] (50). To identify the genes involved in polyphosphate accumulation, we extracted the locus tags of these genes from the annotation files of the genomes of CA23, CU5, A. hydrophila ATCC 7966 (NC_008570.1 and GCA_000014805.1), A. salmonicida A449 (NC_009348.1 and GCA_000196395.1), and A. veronii B565 (NZ_CP012504.1 and GCA_000204115.1).

**(v) Whole-genome sequencing of strains CA23 and CU5.** Genomic DNA of the bacterial isolates was extracted using the Qiagen G20 instrument for bacteria according to the manufacturer’s instructions but with a modification consisting of washing the bacterial pellet with PIV solution (10 ml 1 M Tris-HCl [pH 8] and 58.4 g NaCl in 990 ml of Nanopore water) before resuspension in B1 buffer. The two genomes were sequenced using a PacBio RSII sequencer (Microsynth AG, Switzerland). A PacBio long-read library was assembled with HGAP3 (51), version 2.3.0, and the genomes were annotated using the Prokka pipeline (52), version 1.11.

**(vi) Phylogenetic analysis.** Phylogenetic Analysis and Molecular Evolution (PhaME) software (53) was used to reconstruct the single nucleotide polymorphism (SNP) phylogeny of *Aeromonas* spp. Briefly, PhaME aligned the genomes of *Aeromonas*, removed regions that are not conserved across all genomes, and calculated SNPs from the conserved regions. The SNP alignment was then used to reconstruct maximum-likelihood phylogeny using RAxML v8.2.9 (54). *Aeromonas* genomes were downloaded from GenBank (last accessed on 27 July 2017). The resulting tree was visualized using the Newick utilities (55).

**(vii) Genome comparison.** With the Artemis comparison tool (ACT) (56), a tool for pairwise comparison between complete genome sequences and associated annotations, DNA sequences were used to identify and analyze regions of similarity and difference between genomes. An average nucleotide identity (ANI) calculator from the laboratory of Kostas Konstantinidis (57) (http://enve-omics.ce.gatech.edu/ani/) was used with default settings to measure the nucleotide-level genomic pairwise similarity between the coding regions of the genomes.

### Physiological characterization of CA23 and CU5.

The Vitek 2 Compact consists of biochemical tests for the physiological identification of bacteria. It is an automated microbiology system utilizing growth-based technology under different biochemical conditions contained in colorimetric reagent cards. From a fresh culture of pure bacterial strain, a 0.5 McFarland suspension was prepared using sterile physiological water. The bacterial suspension was then inoculated in wells contained in a Vitek card. Each well corresponds to an individual test substrate. Substrates measure some metabolic activities, such as carbon source utilization, enzymatic activities, and resistance. A transmittance optical system allows interpretation of test reactions using different wavelengths (660 nm, 568 nm, and 430 nm). During incubation at 35.5°C, each test reaction was read every 15 min to measure either turbidity or colored products of substrate metabolism.

Antibiotic resistance testing of CA23 and CU5 was performed using antibiograms containing different antibiotics at defined quantities. The zone diameter (mm) breakpoints were determined using broth microdilution (ISO standard 20776-1; medium, Mueller-Hinton broth; inoculum, 5 × 10^5^ CFU/ml; incubation, sealed panels, air, 35 ± 1°C, 18 ± 2 h) and disk diffusion according to the EUCAST standard method (medium, Mueller-Hinton agar; inoculum, McFarland 0.5; incubation, air, 35 ± 1°C, 18 ± 2 h). The inhibition zone was measured for each antibiotic tested using a Sirscan-2000 instrument.

### Investigation of the pathogenic potential of CA23 and CU5.

The pathogenic potential was investigated using three methods. The first consisted of a classic hemolysis CAMP test. First, the strains were grown on solid Luria-Bertani (LB) medium for 24 h at 30°C, and then the two bacterial strains were inoculated on Columbia agar with 5% sheep blood for 24 h at 37°C. This test determines if bacteria produce β-hemolysin (responsible for blood cell lysis) and/or CAMP factor (enhancement of the hemolysis) (33–35). For this test, Staphylococcus aureus and Streptococcus agalactiae are considered positive controls, while Streptococcus intermedius is the negative control for the hemolytic activity (but positive for the CAMP test).

The second method measures the toxicity of bacteria on the nematode C. elegans (58). *Aeromonas* sp. strains CA23 and CU5 were first grown on LB medium overnight and centrifuged at 2,500 g for 2 min. The supernatant was removed and replaced with a solution of 0.9% NaCl in order to obtain an optical density (OD) of 0.3, 0.5, and 0.7 for each bacterial strain (ODs were measured at 650 nm with an Ultrospec 3100 pro spectrophotometer from Amersham Biosciences). E. coli strain OP50 (OD, 0.7) was used as a negative control. A 384-well plate was used to dispense 100 C. elegans eggs (in 3 μl of M9 medium) along with 10 μl of bacteria (CU5, CA23, or OP50 at a given OD). Dispensing was done using a multidrop Combi dispenser from Thermo Labsystems. Forty-eight replicates were performed for each treatment. C. elegans motility was measured 48 h after dispensing eggs from C. elegans and bacteria in the 384-well plate, using an automated machine vision system measuring worm motility over a duration of 0.3 s. Raw motility scores were measured in each well and normalized with negative controls using Abbott's formula (59). The putative toxicological effect of *Aeromonas* sp. strains CA23 and CU5 was assessed as a reduction of the nematode’s motility relative to that of the control with OP50 only. A decrease in percentage of activity indicated a toxicological effect.

### Inactivation test.

In order to determine an easy method for inactivating the bacterial strains, CA23 and CU5 were cultivated in LB medium overnight. After that, two different methods were tested. First, the liquid cultures of these two bacteria were incubated at 60°C overnight. The second test, performed at room temperature, consisted of mixing the overnight liquid cultures with 23% ethanol under agitation for 10 min or with 35% ethanol for 10 min and overnight agitation. Two hundred microliters of the cultures was then inoculated on solid LB medium and incubated at 30°C. The results were observed after 72 h.

### Accession number(s).

The raw RNA-Seq data were submitted to GenBank under Sequence Read Archive (SRA) accession number SRP153064, while the processed data were submitted in Figshare (https://figshare.com/s/dbf6be9c8286041db24f, https://figshare.com/s/a5cdd9f0de2e04b612b3, https://figshare.com/s/65f3c2f81c4a61620cfd). The complete genomic sequences of CA23 and CU5 were deposited in GenBank under accession numbers NZ_CP023818.1 and NZ_CP023817.1, respectively.

## Supplementary Material

Supplemental file 1
