# Supplementary material for: Remedial Treatment of Corroded Iron Objects by Environmental Aeromonas Isolates
Source: Appl Environ Microbiol. 2019 Jan 23;85(3):e02042-18. doi: 10.1128/AEM.02042-18 (PMC6344634; doi:10.1128/AEM.02042-18)
Supplement: Supplemental file 1 [file 636ffe48afd51fae5c329b3782cbffaa_AEM.02042-18-s0001.pdf]

Supplementary Materials

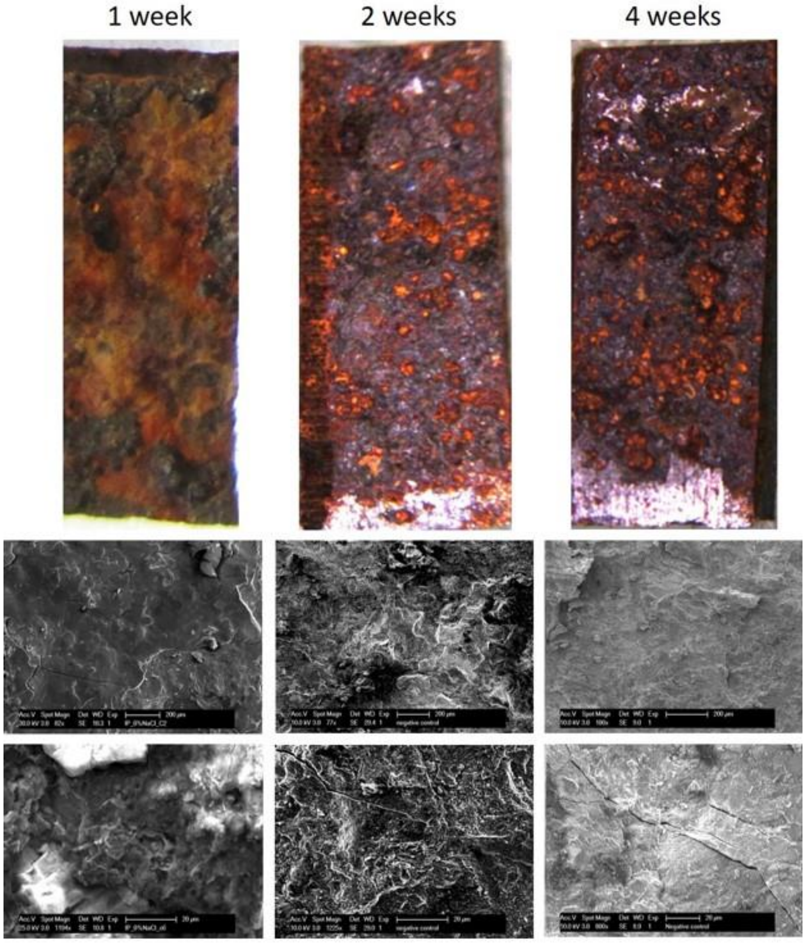

B)

Elements (AT%)	1 week	2 weeks	4 weeks
C	16.45	10.03	11.99
O	57.93	45.03	50.58
Fe	24.58	43.06	33.40
Na	-	-	1.55
Si	0.65	1.88	2.47
S	0.40	-	-

Fig. S1: **Microscopic and spectroscopic analyses of the abiotic control coupons. A)** Visual aspect and scanning electron microscopy (SEM) images of the abiotic control coupons, after 1, 2 and 4 weeks of treatment. **B)** Atomic percentages (AT%) of the elements obtained from the Energy-dispersive X-ray spectroscopy (EDS) measurements performed on the abiotic control coupons after 1, 2 and 4 weeks of treatment. "**-**" stands for not detected.

Table S1: Biochemical reactions of CA23 and CU5 determining their physiological characteristics.

Test	CA23	CU5
Ala-Phe-Pro-Arylamidase (APPA)	-	-
Adonitol (ADO)	-	-
L-Pyrrolidonyl-arylamidase (PyrA)	-	+
L-arabitol (IARL)	-	-
D-cellobiose (dCEL)	-	-
β-galactosidase (BGAL)	-	-
H2S production	-	-
β2S productglucosaminidase (BNAG)	+	+
Glutamyl Arylamidase pNA (AGLTp)	-	-
D-glucose (dGLU)	+	+
γGGlutamyl-transferase (GGT)	-	-
Glucose fermentation (OFF)	+	-
β -glucosidase (BGLU)	-	-
D-maltose (dMAL)	+	+
D-mannitol (dMAN)	-	-
D-mannose (dMNE)	-	+
β -xylosidase (BXYL)	-	-
β(BAlanine arylamidase pNA (BAIap)	-	-
L-Proline-arylamidase (ProA)	+	+
Lipase (LIP)	+	-
Palatinose (PLE)	-	-
Tyrosine-arylamidase (TyrA)	+	+
Urease (URE)	-	-
D-sorbitol (dSor)	-	-
Saccharose/Sucrose (SAC)	+	+
D-tagatose (dTAG)	-	-
D-trehalose (dTRE)	+	+
Sodium citrate (CIT)	-	-
Malonate (MNT)	-	-
5-ceto-D-gluconate (5KG)	-	-
L-lactate alcalinization (ILATk)	-	-
α-glucosidase (AGLU)	-	-
Succinate alcalinization (SUCT)	+	-
β-N-acetyl-galactosaminidase (NAGA)	-	-
α(galactosidase (AGAL)	-	-
Phosphatase (PHOS)	-	-
Glycine arylamidase (GlyA)	-	-
Ornithine decarboxylase (ODC)	-	-
Lysine decarboxylase (LDC)	-	-
L-histidine assimilation (IHISa)	-	-
Courmarate (CMT)	+	+
β(glucuronidase (BGUR)	-	-
O/129 (comp. vibrio.) resistance (O129R)	-	+
Glu-Gly-Arg-arylamidase (GGAA)	-	-
L-malate assimilation (IMLTa)	-	-
Ellman (ELLM)	+	+
L-lactate assimilation (ILATa)	-	-

Table S2: Genes involved in iron reduction in *S. oneidensis* MR-1 (also *pilC* and *pilN* of *G. sulfurreducens*) and their homologues in *A. hydrophila* ATCC 7966, CA23 and CU5 using blastp. Id=identity, cov= coverage, x: not found.

S.oneidensis	**Function**	**Literature**	***A. hydrophila***	**CA23**	**CU5**
cymA	Cytoplasmic membrane-bound periplasmic c-type cytochrome (required for anaerobic respiratory electron transport), is a menaquinol oxidase	(1–3)	*napC*<br>WP_011705485.1<br>(id 31%, cov 95%)	CK910_RS20025<br>(id 31%, cov 95%)<br>WP_043138459.1	CK911_02795<br>(id 32%, cov 95%)<br>WP_098968901.1
undA	Outer membrane 11 heme c-type cytochrome in *Shewanella* sp. strain HRCR-6 and *S. putrefaciens*	(2)	x<br>(ncbi id 22%, low cov)	X<br>(id 37%, cov 67)	x<br>(id 26%, cov 13%)
mtrA	Periplasmic Decaheme c-type cytochrome (electron shuttle). Could function as terminal reductase for soluble Fe(III)	(1–3)	AHA_2765	CK910_RS13415<br>WP_098983641.1	x<br>very low id
mtrB	Outer membrane porin	(1, 2)	AHA_2766	CK910_RS13395<br>WP_098983638.1	x
mtrC	Decaheme c-type cytrochrome	(2, 4)	AHA_2764	CK910_RS13405<br>WP_098983639.1<br>(id 95%)	x<br>very low id<br>(id 31%)
omcA	Outer membrane decaheme c-type cytochrome	(2)	x<br>WP_011706572.1<br>(id 21%, cov97%)	x	x
mtrD	Extracellular respiratory system periplasmic decaheme cytochrome c component	(2, 4)	cytochromeC<br>WP_011706573.1<br>(id55%, cov87%)	cystathionine beta-synthase<br>WP_042866040.1<br>(multispecies: id 51%, cov95%)<br>cytrochrome C<br>WP_098983922.1<br>(id35%, cov71%)	cytochrome C<br>WP_09896950 4.1<br>(id 29%, 87%cov)
mtrE	Extracellular respiratory system outer membrane component	(1)	outer membrane protein (id 27%, cov 98%).<br>WP_011706574.1	hypotheticl protein<br>WP_098983638.1<br>(id 28%, 98%cov)	x
mtrF	Decaheme c-type cytochrome	(1, 3)	x<br>cytochromeC<br>(id 28%, cov 98%)<br>WP_011706572.1	cytochrome C<br>WP_098983639.1<br>(id 24%, cov 98%)	x
cytc 3	Tetraheme c-type cytochrome	(1)	x	x	x

	in the periplasm (electron shuttle between electron carriers)				
menC	Encode o-succinylbenzoic acid synthase, required for Menaquinone biosynthesis (extracellular electron transfer)	(1)	AHA_0528 osuccinylbenzoate synthase WP_011704501.1 (id36%, cov71%)	menC osuccinylbenzoate synthase WP_098980880.1	osuccinylbenzoate WP_098970730.1 (id 36%, cov71%)
menF	Protein for the menaquinone synthesis, isochorismate synthase	(2, 3)	isochorismate synthase WP_011704505.1 id50%, cov90%)	isochorismate synthase WP_098980876.1 (id50%, cov90%)	isochorismate synthase WP_098970723.1 (id51%, cov90%)
hmuZ	FMN binding heme iron utilization protein	Genbank	hutZ heme utilization protein (id 54%, cov 90%) WP_011704904.1	hutZ heme utilization protein (id 54%, cov 90%) WP_098980622.1	hutZ heme utilization protein (id 55%, cov 90%) WP_098968667.1
pilY<br><br><br><br>*mshQ*	TypeIV pili and TypeIV pili adhesion		WP_011704651.1 Pilus biosynthesis protein (id23%, cov54%)<br><br>mshQ WP_011704375.1 (id 37%, cov 97%)	Hypothetical protein WP_098980775.1 (id 39%, cov 97%)<br><br>mshQ WP_098980973.1 (id 39%, cov 88%)	Hypothetical protein WP_098972176.1 (id 39%, cov 97%)<br><br>Hypothetical protein WP_098971018.1 (id 29%, cov 56%)
pilA	Major component of type IV pili (nanowires) in *Geobacter sulfurreducens*		TypeII secretion system WP_011704371.1 (id 47%, cov 34%)	type IV pilin protein WP_098980778.1 (id 59%, cov 24%	type IV pilin protein(id 54%, cov 25%<br><br>WP_098972170.1
fcca	Periplasmic fumarate reductase (genebank)	(2)	Fumarate reductase (id29%, cov 64%) WP_011706984.1	Fumarate reductase (id29%, cov 64%) WP_043555131.1	Fumarate reductase (id29%, cov 74%) WP_042020383.1
hydA	Periplasmic [Fe-Fe] hydrogenase large subunit (genebank)	(2)	x	x	x
pilC geobacter	Type IV pilus inner membrane protein		type II secretion system F family protein (id38%, 98%) WP_011707570.1	type II secretion system F family protein WP_098981882.1 (id39%, 99%)	type II secretion system F family protein (id38%, 99%) WP_068978945.1
pilN geobacter			x	x	x

21

Table S3: Antibiogram results of CA23 and CU5. The bacteria show the same antibiogram profile where there is resistance to ampicillin, amoxicillin and cefalotin. R indicates resistant while S indicates sensitive. The antibiogram was based on Eucast clinical breakpoint tables v.8.0 valid from 2018-01-01.

**Antibiotic tested**	**Disk content (µg)**	**Measured diameter (mm) CA23**	**Results**	**Measured diameter (mm) CU5**	**Result**
Ampicillin	10	**6**	**R**	**11**	**R**
Amoxicillin-clavulanic acid	20-10	**12**	**R**	**15**	**R**
Ticarcillin	75	**16**	**R**	**20**	**R**
Cefalotin	30	**6**	**R**	**13**	**R**
Ceftazidim	10	24	S	20	S
Cefepim	30	25	S	23	S
Ciprofloxacin	5	29	S	26	S
Levofloxacin	5	26	S	21	S
Trimethoprim+Sulfamethoxazol	1.25-23.7	20	S	17	S

A)

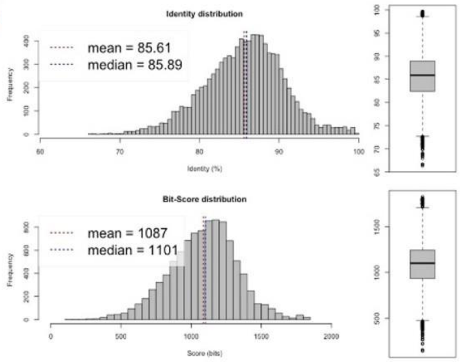

B)

Closest relative to CA23	Isolation site	ANI %
Aeromonas salmonicida CBA100	Skin of rainbow trout, Chile	97.04
Aeromonas bestiarum CECT 4227	nd	97.15
**Closest relative to CU5**	**Isolation site**	**ANI %**
Aeromonas sobria CECT 4245	nd	96.65
Aeromonas sp. EERV15	sand filter	96.34

C)

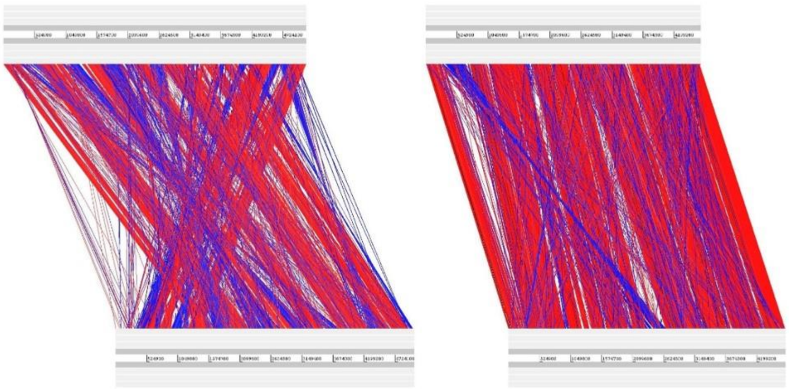

D)

Predicted function	CA23	CU5	*A. hydrophila*	*A. salmonicida*	*A. veronii*
poly-phosphate kinase (*ppk*)	CK910_RS12780 (*ppk1*)<br>CK910_RS19580 (*ppk2*)	CK911_RS04045 (*ppk1*)<br>CK911_RS04810 (*ppk2*)	AHA_2829<br>AHA_1671	ASA_RS07565<br>ASA_RS13405	AMS64_RS14790<br>AMS64_RS15750
Exopolypho-sphatase (*ppx*)	CK910_RS12775	CK911_RS04040	AHA_2830	ASA_RS07560	AMS64_RS15755

Fig. S2: Genomes comparison between CA23 and CU5 and with other *Aeromonas* strains. **A)** ANI results obtained when comparing CA23 and CU5. **B)** Average nucleotide identities (ANI) of CA23 and CU5 and the closest related strains showed in the phylogenomic tree presented in Fig. 2. nd= not determined. **C)** ACT results: CA23 (on the top left) and CU5 (on the top right) are compared respectively with: *A. salmonicida* CBA100 (bottom left) and *A. sobria* CECT 4245 (bottom right). The red and blue bands represent the forward and reverse matches, respectively, the white color represents the absence of matches. **D)** Putative genes involved in polyphosphate metabolism from the genomes of CA23, CU5, *A. hydrophila* ATCC 7966, *A. salmonicida* A449 and *A. veronii* B565.

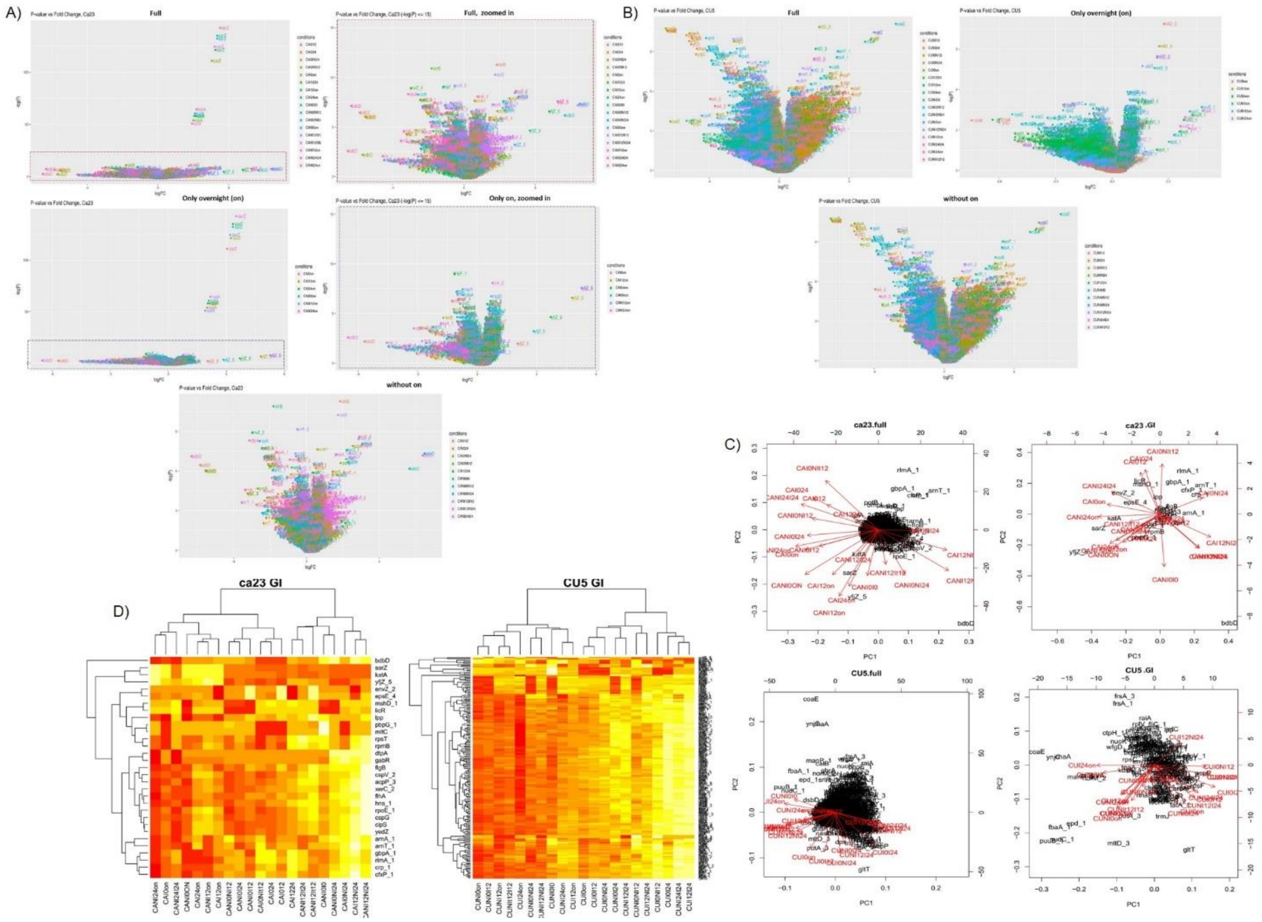

Fig. S3: Transcriptomic analysis of CA23 and CU5. A) and **B)** represent the pairwise comparisons on the Rna-seq data of CA23 and CU5 respectively, using volcano plots (-log10Pvalue, Y axis, logFC, X axis). On the upper line, volcano plots representing the differential gene expression data without any selection. The middle line refers to the comparison of the conditions with and without iron with an overnight culture and the bottom line the pairwise comparison of the conditions with and without iron at three different time points. **C)** Principal Component Analysis (PCA) representation of the differential gene expression data. On the left column the entire data was presented and on the right column the data was filtered according to the |log(FC)|>2.**D)** Comparisons of transcriptional profiles of CA23 and CU5 across samples. Hierarchical clustering of transcripts and samples. Shown is a heatmap showing the relative expression levels of each transcript (columns) in each pairwise comparison (rows). Rows and columns are hierarchically clustered. Each cell in the matrix represents the expression level of a gene feature. Red and white in cells reflect low and high expression levels, respectively. Transcripts that are identified as significantly differentially expressed at |log(FC)|>2.

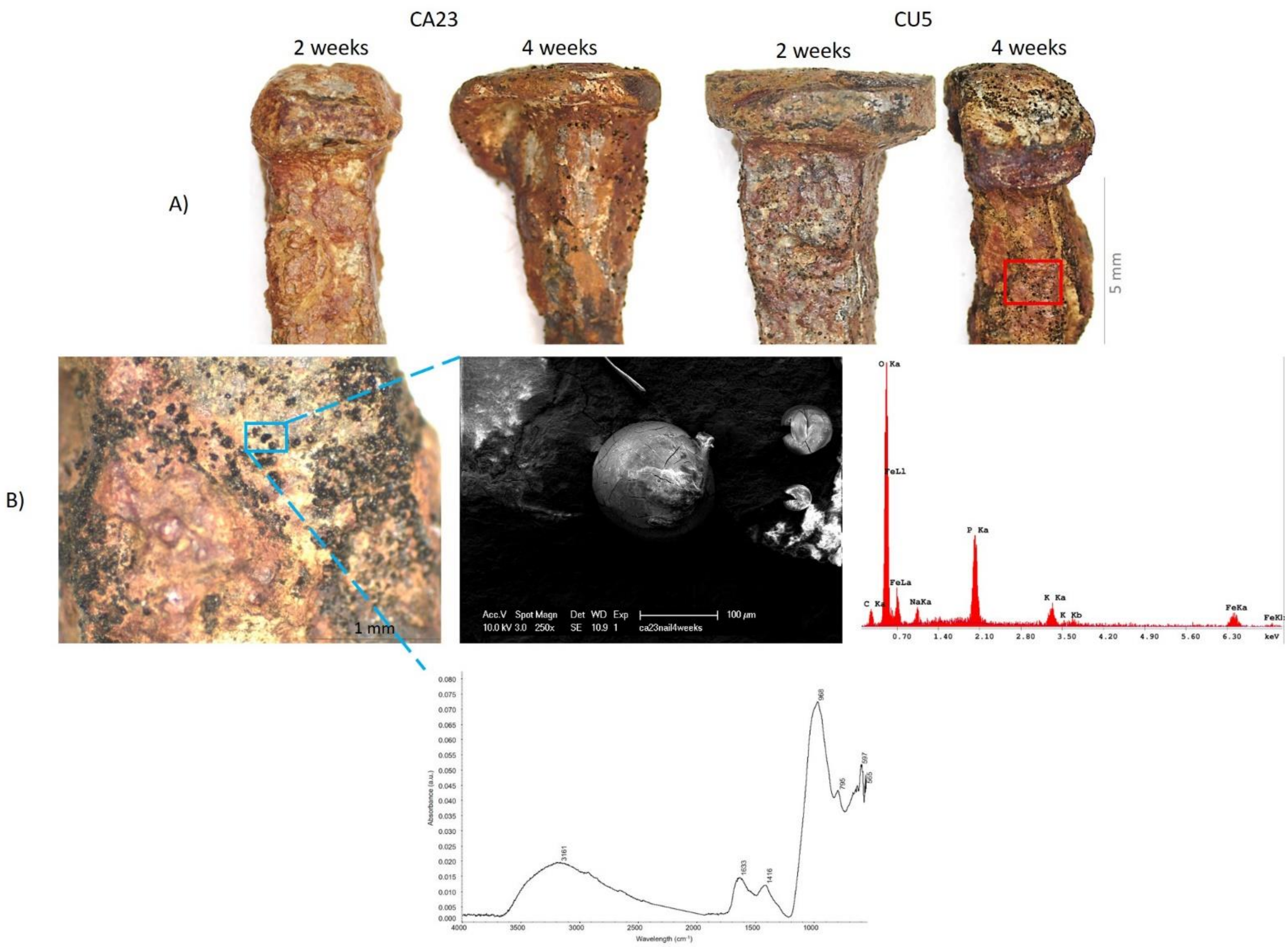

Fig. S4: **First attempt to form biogenic minerals on archaeological iron nails**. **A)** Visual aspect of the iron nails after 2 and 4 weeks of incubation with CA23 and CU5. **B)** Magnified view of the area indicated by a red square on the upper right nail (CU5/4weeks). The scanning electron microscopy (SEM) image, Energy Dispersive X-ray Spectroscopy (EDS) and Fourier Transform InfraRed (FTIR) spectra corresponding to the area indicated by a blue square on the magnified view.

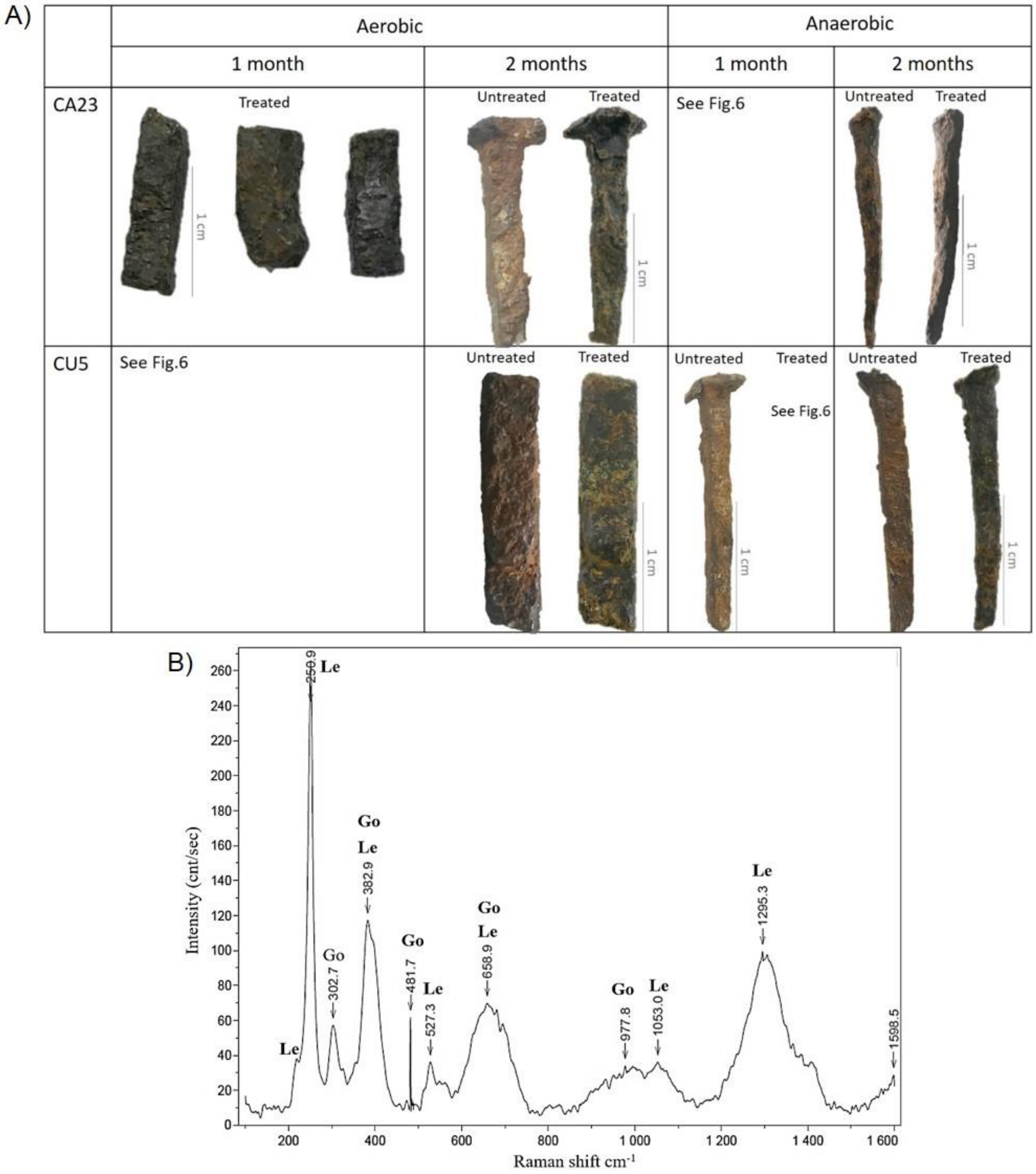

Fig. S5: **Visual observations and Raman analysis of archaeological iron objects**. **A)** Photos of the objects before treatment (untreated) and after being aerobically or anaerobically treated with CA23 and CU5. **B)** Raman spectrum obtained on the abiotic control (an archaeological iron object) after 1 month of incubation in the gel delivery system (without bacteria). Main corrosion compounds were identified as goethite (Go) and lepidocrocite (Le).

References

1. Lies DP, Mielke RE, Gralnick JA, Newman DK. 2005. *Shewanella oneidensis* MR-1 Uses overlapping Pathways for Iron Reduction at a Distance and by Direct Contact under Conditions Relevant for Biofilms. Appl Environ Microbiol 71:4414–4426.
2. Saffarini D, Brockman K, Beliaev A, Bouhenni R, Shirodkar S. 2015. Shewanella oneidensis and extracellular electron transfer to metal oxides, p. 21–40. *In* Bacteria-Metal Interactions.
3. Weber K a., Achenbach L a., Coates JD. 2006. Microorganisms pumping iron: anaerobic microbial iron oxidation and reduction. Nat Rev Microbiol 4:752–764.
4. Cheng YY, Li BB, Li DB, Chen JJ, Li WW, Tong ZH, Wu C, Yu HQ. 2013. Promotion of iron oxide reduction and extracellular electron transfer in *Shewanella oneidensis* by DMSO. PLoS One 8.
